# Cellular senescence is a promising target for chronic wounds: a comprehensive review

**DOI:** 10.1093/burnst/tkaa021

**Published:** 2020-06-23

**Authors:** Ziwen Wang, Chunmeng Shi

**Affiliations:** Institute of Rocket Force Medicine, State Key Laboratory of Trauma, Burns and Combined Injury, Third Military Medical University (Army Medical University), No. 30 Gaotanyan Street, Chongqing, 400038, China

**Keywords:** Chronic wounds, Cell senescence, Therapeutic agent, Skin, Mucosa

## Abstract

Chronic wounds include, but are not limited to, radiation ulcers, pressure ulcers, vascular ulcers and diabetic foot ulcers. These chronic wounds can persist for years without healing and severe ulcers may lead to amputation. Unfortunately, the underlying pathologies of refractory chronic wounds are not fully characterized, and new treatments are urgently needed. Recently, increasing evidence has indicated that cell senescence plays an important role in the development of chronic wounds, and preventing cell senescence or removing senescent cells holds promise as a new therapeutic strategy. In this review, we aim to probe these latest findings to promote the understanding of cellular senescence in the pathological process and potential management of chronic wounds.

## Background

Chronic wounds do not progress in a timely manner during the healing process, causing a huge financial and medical burden on the health system [1, 2]. Chronic wounds can be classified as radiation ulcers and non-radiation ulcers, including pressure ulcers, diabetic foot ulcers and vascular ulcers (including venous and arterial ulcers) [3]. These chronic wounds can last for several months to years and often recur, leading to functional loss of skin or mucosa and decreased life quality [4]. Various prevention and treatment approaches, such as anti-inflammatory drugs, growth factors, local anaesthetics, extracellular matrix (ECM) treatment, negative-pressure wound therapy and engineered skin have been used to cure chronic wounds, but many of these therapies are less effective [5, 6]. Therefore, new, valid agents or treatments are urgently needed.

The common features of these wounds include persistent infection, prolonged or exaggerated inflammation, failure of epidermal and/or dermal cells to respond to repair stimuli and the formation of biofilms caused by resistant microorganisms [3, 7]. Thus, these pathophysiological phenomena contribute to the failure of wound healing, but the underlying pathologies are numerous or even unclear in different chronic wounds. Recent evidence has shown that senescent cells accumulate in some chronic wounds, promoting the development of poorly healing wounds [8–12]. Furthermore, removing senescent cells or preventing cell senescence has been reported to mitigate chronic wounds [11, 12]. Based on these new advances, we hypothesize that cellular senescence is a promising target for chronic wounds.

## Review

### Clinical challenges of chronic ulcers

Wound healing is among the most complex processes in the human body [13, 14]. At the cellular level, wound healing requires the participation of many cell types, including fibroblasts, keratinocytes, macrophages, endothelial cells and platelets that are timely coordinated in space [15]. The physiological process of wound healing can be divided into four phases: haemostasis, inflammation, proliferation and remodelling [16, 17]. After injury, the clotting cascade is activated immediately and haemostasis occurs, preventing blood loss and providing a temporary matrix for cell migration [18]. During this process, immune cells, fibroblasts and endothelial cells are attracted by several growth factors, such as platelet-derived growth factor (PDGF), transforming growth factor-β and epidermal growth factor (EGF), which are secreted by platelets and can activate the healing process. Meanwhile, inflammatory cells migrate to the wound site and remove bacteria or necrotic tissues. Next, macrophages can release many growth factors and cytokines that can initiate the formation of granulation tissue. Next, fibroblast growth factor (FGF) and vascular endothelial growth factor stimulate endothelial cells to proliferate; FGF, transforming growth factor-α and EGF promote epithelial cells to proliferate and migrate, increasing the formation of blood vessels and epithelialization. Finally, organized collagen bundles are remodelled by the provisional matrix when the wound has closed, keratinocytes begin to differentiate and stratify and scar remodelling appears; this phase may last for 1–2 years or longer [19].

**Figure 1.**
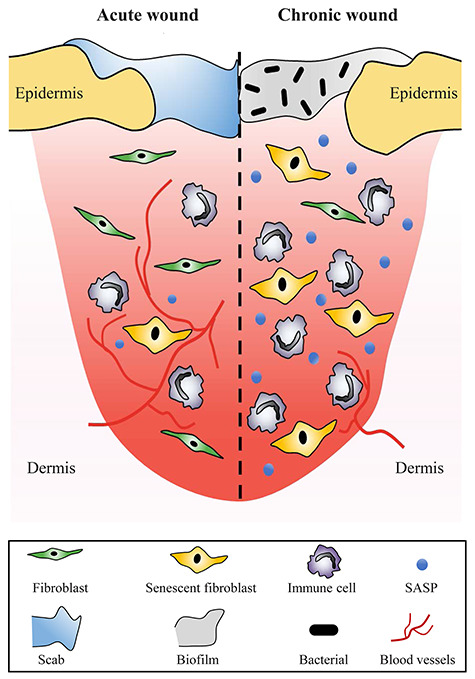
Molecular and cellular differences between chronic and acute wounds. The transient inflammatory response is initiated in the healing process of acute wounds and provides a beneficial environment for re-epithelialization and regeneration. However, chronic wounds stall in the inflammatory phase, leading to persistent inflammation. Chronic wounds exhibit the accumulation of senescent cells and an increased senescence-associated secretory phenotype (SASP) with poor blood vessel infiltration

In most clinical settings, wound healing can be properly executed with highly organized and coordinated cellular processes, and many acute wounds can heal [6]. However, if healing cannot progress in an orderly and timely manner, chronic wounds may form [20]. The nonhealing state results in the loss of function and morbidity and has a huge impact on life quality [13]. The 5-year mortality rate after amputation is approximately 50% [21, 22]. In general, chronic wounds stall in the inflammatory phase, leading to persistent inflammation. Although the aetiology is different at the molecular level, chronic wounds have some prevalent characteristics, such as increased proteases, pro-inflammatory cytokines, harmful reactive oxygen species (ROS), prolonged infection, accumulation of senescent cells and dysfunctional stem cells. Additionally, PDGF and microorganisms stimulate the constant influx of immune cells that contribute to the amplified and persistent pro-inflammatory cytokine cascade with high levels of protease. In chronic wounds, the level of proteases is too high and cannot be suppressed to normal levels, resulting in ECM destruction and promotion of the degradation of growth factors and their receptors. However, in acute wounds, the inhibitors of proteases can strictly control ECM levels. When the ECM is proteolytically destroyed, the wound fails to move on to the proliferative phase and attracts many inflammatory cells, amplifying the inflammatory cycle (Figure 1) [23].

Numerous topical dressings and antimicrobials are available for clinicians. However, few prospective studies have favoured their effectiveness in promoting chronic wound repair, and doctors tend to use strategies based on personal experience. Many products or therapies, such as the Oasis wound matrix, Promogran, Renasys, Regranex, OxyHeal1000 and Integra, have been applied to cure chronic wounds but the effect is not very ideal and the treatment duration is relatively long. Bioengineered substitutes containing living cells (including Grafix, Dermagraft and Apligraf) have been developed to increase the effect on the skin [13]. Additionally, cellular therapies are effective and safe in treating chronic wounds for people with diabetes and other impaired conditions. However, despite using the best care, 15–20% of all chronic wound sufferers show a poor response to the therapies mentioned above.

It is crucial not only to improve the symptoms of the wound, such as pus and pain, but also to ameliorate potential metabolic and systemic disorders, such as peripheral arterial disease and infections. More importantly, the choice of treatments should be based on the available evidence to ensure the highest possible efficacy. Many challenges exist to study the mechanisms and main contributing factors of chronic wounds, including complex fundamental changes and various processes or cell types involved in chronic wounds, as well as the lack of a specific target on which to focus interventions in this multifactorial system. Additionally, it is essential to further understand the physiological perturbations and underlying molecular mechanisms in nonhealing wounds; however, it would be challenging to establish an optimal animal model to replicate their complexity [24].

### Characteristics of cell senescence and the senescence-associated secretory phenotype

Cell senescence is a regulatory response to multiple types of cellular stress, such as DNA damage, telomere erosion, oncogene activation, oxidative damage, protein misfolding and exposure to extracellular signals (like mitogens and cytokines), which may occur at any point in the cell’s life cycle [25, 26]. At the molecular level, p53 and p16^INK4a^/Rb are two core senescence-regulating pathways in cellular growth arrest [19]. During this process, cells undergo a series of phenotypic transformations with prolonged cell cycle arrest. There is increased production of ROS, persistent DNA damage foci (containing DNA damage sensors such as gamma histone variant H2AX and serine/threonine-kinase Ataxia Telangiectasia Mutated (ATM)-like protein, which are dependent on the stimulus and frequency) and epigenetic rearrangements in senescent cells. After injury, excessive ROS in the wounds can destroy proteins, lipids and nucleic acids, contributing to impaired stem cell function and cell senescence [27]. These cells fail to activate and expand, undergoing accelerated entry into a full senescence state, even in a youthful environment [28]. Additionally, telomere shortening can limit the proliferation of primary cells to a finite number of divisions after injury, resulting in replicative senescence; meanwhile, the deficiency of growth factors also contributes to premature senescence at the wound site [29, 30]. Moreover, mechanical trauma or radiation promotes DNA double- and single-strand breaks, which are known inducers of cell cycle arrest signals [31]. Therefore, cell senescence is a common phenomenon following injury.

Cell senescence is another fate besides apoptosis when cells are exposed to irreparable or excessive cellular and genotoxic stress, and senescent cells can undergo apoptosis resistance [25]. Furthermore, senescent cells are highly metabolically active in tissues; they can secrete high levels of senescence-associated secretory phenotype (SASP) components, including cytokines, matrix remodelling proteins and growth factors [32]. These molecules can change the microenvironment and play an important role in a wide range of biological processes from physiology to pathology [33, 34]. Additionally, these secreted factors cause inflammation, which may be crucial for the removal of senescent cells by phagocytosis, at least in some cases; for example, inflammation can drive the recruitment and activation of immune cells, including monocytes/macrophages, natural killer cells and T-cells, leading to the subsequent elimination of senescent cells [35–37]. SASP components also trigger growth arrest and dysfunction in neighbouring cells via a mechanism that generates DNA damage and ROS in a paracrine manner [38–40]. There is a distinct hierarchy among SASP factors—some of them are necessary for maintenance, and others are used to induce a secretory phenotype. The expression of interleukin-1α can activate the C/EBPβ and NF-κB pathways, which cooperatively regulate SASP components in various senescence contexts and result in the induction of the SASP [38, 41, 42]. Other factors, such as interleukin-6 and chemokine receptor 2-binding chemokines, can form positive feedback loops that reinforce the expression of the SASP as well as growth arrest [42, 43]. Therefore, the SASP has powerful paracrine and autocrine activities, which could create an inflammatory and profibrotic microenvironment.

### Cell senescence in wound healing and regeneration

The role of cell senescence has been mainly limited to cellular damage or stress. However, cell senescence has been observed in human, chicken, mouse and quail embryo development [44–46], suggesting that it is a conservative characteristic of vertebrate embryonic development. In addition to embryonic development, cell senescence also occurs in adults in physiologically programmed ways; particularly, placental syncytiotrophoblasts and normal megakaryocytes undergo cell senescence as part of the natural maturity programme [47, 48]. Remarkably, however, senescent cells were ultimately eliminated in both normal development and physiology processes that involve delayed infiltration of macrophages and compensatory apoptosis [36, 37, 44, 46, 49]. Additionally, potent and rapid activation of cell senescence in adult animals has been identified in multiple-wound-healing models.

Activation of the p16^INK4a^ promoter is observed within 2–3 days in injured tissues, peaks between 4 and 7 days and then resolves over 2–3 weeks using p16^INK4a^ reporter mice [50]. Significant induction of cellular senescence occurs during salamander limb regeneration, but rapid and effective mechanisms of senescent cell clearance operate in normal and regenerating tissues. Cellular senescence is a normal process during salamander limb regeneration and it is subject to dynamic regulation [51]. The expression of SASP cytokines and NF-κB activation are found at the wound sites and the clearance of p16^INK4a^-positive cells delays wound closure with increased fibrosis, suggesting cell senescence is crucial for optimal healing [50]. After the stage of cell proliferation and ECM deposition, myofibroblasts from the wound become senescent, with cell cycle arrest and upregulation of the ECM-degrading enzyme, emphasizing the importance of cell senescence as a limiting mechanism of fibrosis in wound healing [50]. However, animals deficient in p16^INK4a^ show no defects in the healing process, indicating that not p16^INK4a^, but some feature of p16^INK4a^-expressing cells (senescent cells), promotes tissue remodelling in the wound [52]. Senescent endothelial cells and fibroblasts are induced instantaneously at the wound site, where they promote wound closure by inducing myofibroblast differentiation by secreting platelet-derived growth factor alpha polypeptide a (PDGF AA); therefore, it is probable that SASP components are candidates for this effect [50]. Furthermore, matricellular protein cellular communication network factor 1 is dynamically expressed following injury and could activate the ROS-dependent p16^INK4a^/pRb pathway, contributing to the expression of antifibrotic genes and cellular senescence in the wound, where the accumulation of senescent fibroblasts in granulation tissues and expression of antifibrotic genes in the healing cutaneous wounds are observed [53]. Maintaining the integrity of the tissues around the wound is a key aspect of wound healing and this process depends on ECM deposition, which should be strictly controlled; otherwise, it will lead to fibrosis and scarring [54]. More importantly, there is convincing evidence that cells undergoing injury-induced senescence are usually cleared through immune-mediated removal in the late wound-healing process [36, 37, 49]. Taken together, the results of these studies show that cell senescence promotes skin development, repair and regeneration in the early stages of wound healing.

### Cell senescence in the impaired healing of chronic wounds

**Figure 2.**
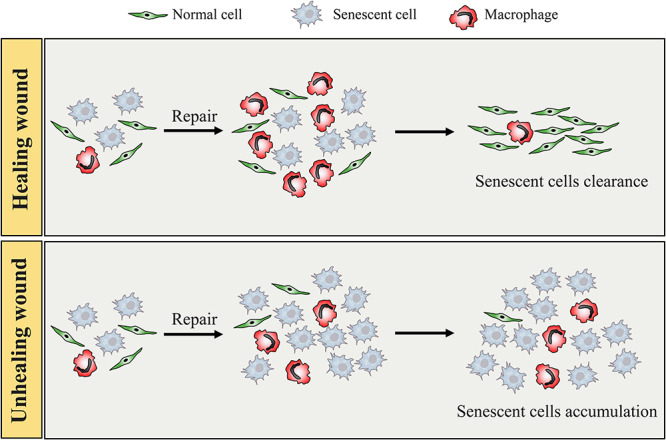
Cell senescence acts as a double-edged sword in wound healing. Cell senescence is crucial for the optimal healing process of acute wounds at the early stage, and then senescent cells are cleared by macrophage-dependent immunosurveillance. However, reduced chemotaxis of macrophages in chronic wounds is involved in the impaired capacity to migrate to the sites where senescent cells accumulate (e.g. impaired response to senescence-associated secretory phenotype factors), contributing to the accumulation of senescent cells. Additionally, senescent cells induce a pro-senescent and pro-inflammatory environment, and the process of cell senescence is constantly being amplified in chronic wounds

Senescent cells secrete a series of SASPs that regulate the surrounding microenvironment, which directly or indirectly affects various processes of regeneration, including angiogenesis, matrix remodelling, cell plasticity and growth [42, 43, 55]. Senescent cells are then cleared by macrophage-dependent immunosurveillance. However, senescent cells are not removed from chronic wounds, causing persistently elevated secretion of cytokines and decreased proliferation [56]. There is a shift to type M2 macrophages during ageing and trauma that correlates with a reduced immune response, tumour promotion and impaired phagocytosis and chemotaxis [57, 58]; and it is speculated that reduced chemotaxis of macrophages in chronic wounds is involved in the impaired capacity to migrate to the places where senescent cells accumulate (e.g. impaired response to SASP factors), contributing to the accumulation of senescent cells [59]. Because macrophages are key immune cells for the clearance of senescent cells, their absence should lead to high levels of senescence markers (unless other compensatory pathways are activated) and SASPs with sustained inflammation (Figure 2). Additionally, fibroblasts from chronic wounds are less able to respond to growth factors that usually stimulate mitotic responses. Studies have shown that the responses to FGF, EGF and PDGF are reduced in senescent cells, activities that are not related to the reduction in the number of receptors but could be due to the inactivation of intracellular signals [60]. Elevated matrix metalloproteinase levels are observed in chronic wounds and have been implicated in the degradation of growth factors and delays to wound healing [9]. More importantly, to identify accurate therapeutic strategies to remove the senescent cells and their products, differences in the SASP between healing wounds and chronic wounds need to be investigated.

In the radiation-induced chronic wound model, we reported that senescent cells and DNA damage accumulate in radiation-induced ulcers in both animal and human tissues [11, 12], and the development of radiation ulcers is accelerated when senescent cells are injected subcutaneously into the irradiated area [11, 12]. Skin injected with senescent cells shows an accelerated process of redness, swelling, hair loss and ulceration. In non-radiation-induced chronic wounds, senescent fibroblasts, endothelial cells and keratinocytes have been reported to accumulate at the wound sites [9, 61–63]. Senescent cells with a prolonged inflammatory response, niche disruption or progenitor depletion have been reported in non-healing pressure ulcers, resulting in impaired wound healing [64]. In venous hypertension, premature cell senescence was observed to result in venous ulcers with delayed healing: approximately 15% of senescent cells were isolated from the wound sites and the rate of wound healing was negatively correlated with the number of senescent cells [65]. The SASP in chronic wounds can result in oxidative stress, which contributes to abnormal metabolic changes and DNA damage in patients with diabetes [9, 66]. Additionally, a long-term inflammatory response may have adverse effects on wound closure. Long-term exposure to chronic wound fluid may also decrease cell activity in the wound and lead to cell senescence. Moreover, prolonged inflammation and cell senescence may have adverse effects on the efficacy of topical biologics (including growth factors) by creating an environment with fewer receptors for growth factors [8]. Those phenomena indicate that cell senescence plays a vital role in both radiation-induced and non-radiation-induced chronic ulcer development.

Stem cell proliferation and signal transduction occur throughout each stage of wound healing; thus, stem cell dysfunction can lead to chronic wounds [67]. Cell-based therapy is a distinct and reasonable step to treat chronic wounds, and its clinical application may be beneficial because stem cells can directly interact with the environment in multifactorial and complex ways at the wound sites and they can directly differentiate and replace components of the lost tissues or cells, such as fibroblasts, keratinocytes and skin appendages. Additionally, stem cells possess powerful immunomodulatory properties and can activate various cytoprotective genes in target tissues [68]. Furthermore, mesenchymal stem cells have been characterized to play a vital role in the healing process [69, 70]; when injury occurs, they can be recruited into the circulation and engraft into the remodelling microvasculature. However, the function of stem cells in chronic wounds is defective [69, 71, 72]. Endothelial progenitor cells from patients with diabetes can adhere to tumour necrosis factor-activated endothelial cells and show damaged migration capacity to wound sites [67]. The currently available evidence also indicates that persistent senescent cells delay the healing of chronic wounds; senescent cells also induce a DNA damage response and cell senescence in neighbouring cells via processes involving ROS and gap junction-mediated cell–cell contact [40]. Senescent cells were transplanted into the skeletal skin and muscle of immunocompromised neuron-specific gene (NSG) mice and, 3 weeks after the last transplantation, the dermal fibroblasts and myofibres expressed various senescence markers around the area where senescent cells were transplanted but not in the area with non-senescent or no cells injected [73]. Therefore, resident senescent cells can result in the dysfunction of stem cells in the healing process of chronic wounds. In this regard, senescent cells may contribute to the dysfunction of stem cells and delay the healing process of chronic wounds. To conclude, abnormal wound healing is closely linked to an impaired microenvironment, biofilm deposition and cell function, and cell senescence is detrimental in the healing process of chronic wounds.

### Senescent cells as an emerging therapeutic target for chronic wounds

Very recently, several studies have shown that the clearance of endogenous senescent cells or the prevention of cell senescence could be a beneficial repair process in chronic wounds. To screen candidate compounds that can ameliorate cell senescence and prevent radiation ulcers, we have established a cell senescence model induced by radiation *in vitro* using fibroblasts, because they play a crucial role in ulcer development [11, 12]. We also established three ulcer models, for skin ulcers, intestine ulcers and oral mucositis, to verify the effectiveness of the screened drug [11]. Next, we identified a natural nucleoside analogue compound, cordycepin, which can prevent cell senescence and radiation ulcer effectively using the small-molecule library we established before [11]. Additionally, dasatinib + quercetin (DQ) has been reported to selectively promote the apoptosis of senescent cells [74, 75]. We identified that senescent cells are removed by DQ by inducing senescent cell apoptosis directly *in vivo* and *in vitro*; not surprisingly, DQ treatment also alleviates radiation-induced ulcers [12]. Moreover, our findings suggest that cordycepin can directly bind to adenosine 5′-monophosphate-activated protein kinase (AMPK) near the autoinhibitory domain at the α1 and γ1 subunits, relieving the autoinhibition of AMPK and promoting the translocation of nuclear factor E2-related factor 2 (NRF2) to the nucleus [11]. Furthermore, activation of NRF2 or AMPK can be a therapeutic target to prevent cell senescence and radiation ulcers, providing a reference for future drug development. Similarly, it was reported that rapamycin can prevent epithelial stem cell senescence by inhibiting the mammalian target of rapamycin and protecting against radiation-induced mucositis [76]. In non-radiation-induced chronic wounds, a link between ageing and fibrosis has also been discovered during skeletal muscle injury, and inactivation of the endocytic adapter Numb in mice leads to sustained p53-dependent senescence of myofibroblasts after severe injury, leading to reduced regeneration potential [77]. The regenerative capacity in Numb mutants is functionally rescued and the levels of cell senescence markers are reduced to normal levels by p53 ablation or antioxidant treatment [77]. However, it should be noted that irradiation induces ROS and DNA damage in cells, resulting in cell apoptosis or senescence, which is more complicated than non-radiation-induced cell senescence. The therapy of senescent cell clearance and prevention in radiation-induced chronic wounds is more challenging than that in non-radiation-induced chronic wounds. We should identify accurate therapeutic strategies or agents to prevent cell senescence and clear senescent cells for different wounds. Thus, preventing cell senescence and removing senescent cells are emerging therapeutic strategies for chronic wounds.

## Conclusions

Chronic wounds are a huge challenge for wound-care researchers and clinicians. The use of advanced treatment modalities, such as tissue replacement and growth factors, may offer a strategy to accelerate wound closure in chronic wounds; however, some wounds still show no response to these treatments. Senescent cells accumulate in chronic wounds, creating an environment of prolonged inflammation and contributing to the dysfunction of stem cells. Increasing evidence has shown that preventing cell senescence or removing senescent cells can mitigate chronic wounds, and cellular senescence can be a promising target for chronic wounds. Although some clues have been provided in this review, the underlying mechanism of senescent cells contributing to the development of chronic wounds requires further investigation. More importantly, more research is needed regarding cell senescence in chronic ulcers, as well as the evaluation of the clinical significance of this strategy.

## Abbreviations

ECM: extracellular matrix; PDGF: platelet-derived growth factor; EGF: epidermal growth factor; FGF: fibroblast growth factor; ROS: reactive oxygen species; SASP: senescence-associated secretory phenotype; DQ: dasatinib + quercetin; AMPK: adenosine 5′-monophosphate-activated protein kinase; NRF2: nuclear factor E2-related factor 2

## Data Availability

Not applicable.
